# Evaluation of drug-targetable genes by defining modes of abnormality in gene expression

**DOI:** 10.1038/srep13576

**Published:** 2015-09-04

**Authors:** Junseong Park, Jungsul Lee, Chulhee Choi

**Affiliations:** 1 Department of Bio and Brain Engineering, KAIST, Daejeon, 305-701, Republic of Korea; 2 KAIST Institute for the BioCentury, KAIST, Daejeon, 305-701, Republic of Korea

## Abstract

In the post-genomic era, many researchers have taken a systematic approach to identifying abnormal genes associated with various diseases. However, the gold standard has not been established, and most of these abnormalities are difficult to be rehabilitated in real clinical settings. In addition to identifying abnormal genes, for a practical purpose, it is necessary to investigate abnormality diversity. In this context, this study is aimed to demonstrate simply restorable genes as useful drug targets. We devised the concept of “drug targetability” to evaluate several different modes of abnormal genes by predicting events after drug treatment. As a representative example, we applied our method to breast cancer. Computationally, PTPRF, PRKAR2B, MAP4K3, and RICTOR were calculated as highly drug-targetable genes for breast cancer. After knockdown of these top-ranked genes (i.e., high drug targetability) using siRNA, our predictions were validated by cell death and migration assays. Moreover, inhibition of RICTOR or PTPRF was expected to prolong lifespan of breast cancer patients according to patient information annotated in microarray data. We anticipate that our method can be widely applied to elaborate selection of novel drug targets, and, ultimately, to improve the efficacy of disease treatment.

Systematic identification of novel drug targets is one of the most common applications of high-throughput expression profiles. As one of routine methods, differentially expressed genes (DEGs), primarily obtained from microarray experiments, have been explored. However, the notorious inconsistency and low reproducibility of microarray data require large sample sizes and limit practical use of DEGs for this purpose^1^,^2^,^3^. Moreover, gene expression levels of groups are often not significantly different, and most DEGs are not obviously associated with phenotypes^4^. DEGs might be good biomarkers for certain phenotype, but it is not ensured that they can be used as drug targets. Despite application of diverse normalization, resampling, and gene-set approaches^5^,^6^,^7^, analysis of expression levels alone is not sufficient to identify good drug targets. Therefore, novel methods are required to incorporate features other than expression differences.

Previously, we used transcriptional responses to develop a platform to identify phenotype deterministic genes, and we successfully identified several causative genes responsible for chemo-sensitivity to tamoxifen and epirubicin^8^. Vandin *et al.*^9^ and Merid *et al.*^10^ also developed methods to distinguish driver and passenger mutations in cancer genomes. Even though abnormal driver genes were identified, however, repairing them is not always possible. There is no clear understanding of working mechanism for many of these molecules, and several trials to targeting them eventually failed^11^. Diverse drug resistance mechanisms^12^, or discrepancy between preclinical tumor models and patients^13^,^14^, may be responsible for these unsuccessful examples. Therefore, simply identifying abnormal genes is not sufficient for their use as effective drug targets. Zaman *et al.* predicted effective breast cancer subtype-specific drug targets, which were evaluated by integrated sequencing and functional RNAi screening data. They identified genes that are essential for cell proliferation and survival, and overlaid this information onto human signaling network to represent core-signaling network^15^. In addition, there are several studies or databases using functional cancer genomics, each of which performed massive RNAi screening to identify genes that are required for cell proliferation or viability^16^,^17^,^18^,^19^. These projects systematically examined cancer cell line-specific genetic dependencies onto cell viability or proliferation, but still lacks the way to restore normality. Moreover, cancer shows diverse functional hallmarks other than proliferation, some of which are most deterministic factors compared to normal samples. In this regard, comparisons with normal counterpart are necessary, and various patterns of abnormality in gene expression and regulation should be dissected.

We established the concept of “drug targetability” and applied it to identify highly drug targetable genes in breast cancer. Among many modes of abnormality in gene expression, simply restorable genes should be targeted to recover normal phenotypes. Because most drugs reduce activity or expression of certain genes^20^,^21^, we defined drug targetability as the degree of similarity with normal samples after inhibition of abnormal genes. To validate our computational predictions, we performed cell death and migration assays following knockdown of top-ranked genes (high drug targetability) using siRNA. Successful results suggest that the method we propose here can be widely applied to elaborate selection of novel drug targets for various diseases.

## Results

### Overview of the approach

**Figure 1:**
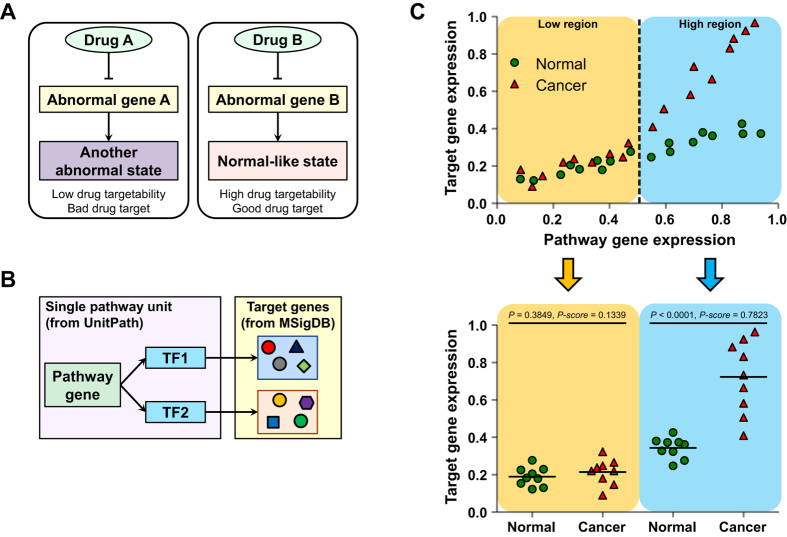
Overview of the study approach. (**A**) Several abnormal genes (gene A and B) can induce disease conditions. When abnormal gene A is inhibited by drug A, a phenotype of patient is converted to another abnormal state (left, indicating low drug targetability and bad drug target). In contrast, inhibition of abnormal gene B with drug B lead to normal-like state (right, indicating high drug targetability and good drug target). (**B**) For a gene involved in a pathway unit (pathway gene), target genes of TFs involved in the same pathway unit were considered to be target genes of the pathway genes. UnitPath database was used for pathway information, and MSigDB was used to retrieve target gene information of TFs. (**C**) Schematic diagram of the evaluation of P-score in the low region (LP) and that in the high region (HP). According to the level of pathway gene expression (x-axis), we divided the low region (≤0.5) and high region (>0.5), and then compared expression levels of the target genes (y-axis) for each region.

Associated with disease symptoms, many genes have abnormal expressional profiles and transcriptional responses compared to the control. To identify novel drug targets among these genes, we should select those restorable after drug treatment. Since the most frequently used drugs (e.g., monoclonal antibody and chemical inhibitor) reduce the expression or activity of targeted genes, we defined drug targetability to reflect this attribute among several modes of abnormality (Fig. 1A). Similar to our previous report^8^, we considered genes in the same pathway units with transcription factors (TFs) as genes that can modulate transcriptional responses. We evaluated all signaling molecules in pathway units of the UnitPath database and referred to them as pathway genes. We considered target genes of the TFs to be transcriptionally controlled by each pathway gene (target genes of the pathway gene, Fig. 1B). We included 1,191 pathway genes and 10,305 target genes of pathway genes in the analysis.

**Figure 2:**
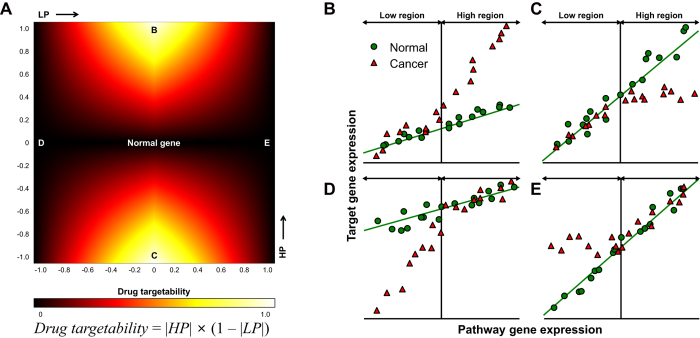
Calculation of drug targetability and different modes of abnormality. (**A**) Equation and its two-dimensional heat map of drug targetability in terms of LP and HP. (**B**–**E**) Diverse modes of abnormality in gene expression for the cases indicated in (**A**). (**B**,**C**) represent behaviors of good drug targets. (**D**,**E**) shows likely examples of bad drug targets.

For all pathway gene-target gene pairs, we plotted corresponding expression levels, and then distinguished low regions and high regions according to the pathway gene expression levels: pathway gene with expression level larger than 0.5, high region; otherwise (≤0.5), low region. We compared expression levels of target genes in two groups (e.g., normal and cancer) for each region separately, and calculated the P-score using the *P*-value as described in the *Materials and Methods* (Equation (2) and Supplementary Fig. S1). As shown in the example of Fig. 1C, if one target gene has a significantly higher expression level in cancer than the control (high region, blue), the P-score in the high region (HP) is large. If one target gene has a similar expression level in cancer and normal (low region, yellow), the P-score in the low region (LP) is small. We defined drug targetability to have a large value when the HP is large and the LP is small, as following:
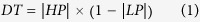


Figure 2A presents the defined equation and its corresponding heat map of drug targetability. According to the definition, the center region on the heat map (both LP and HP have very small values) indicates normal genes in terms of transcriptional response. In the B and C regions on the heat map (small LP and large LP), target gene expression was significantly different compared to the control in the high region, but was similar in the low region (Fig. 2B,C). As a result, we can expect that drugs targeting or inhibiting the pathway gene will restore its transcriptional response similarly with a normal phenotype. In the D and E regions on the heat map (large LP and small HP), however, target gene expression was significantly different only in the low region (Fig. 2D,E). For these cases, suppression of the pathway gene is unlikely to restore normality. As shown in these examples, we classified several modes of abnormality by evaluating drug targetability for all pathway gene-target gene pairs. Drug targetability was defined to have a large value when suppression of the gene was expected to restore transcriptional normality.

### Evaluation of drug targetability in breast cancer

**Figure 3:**
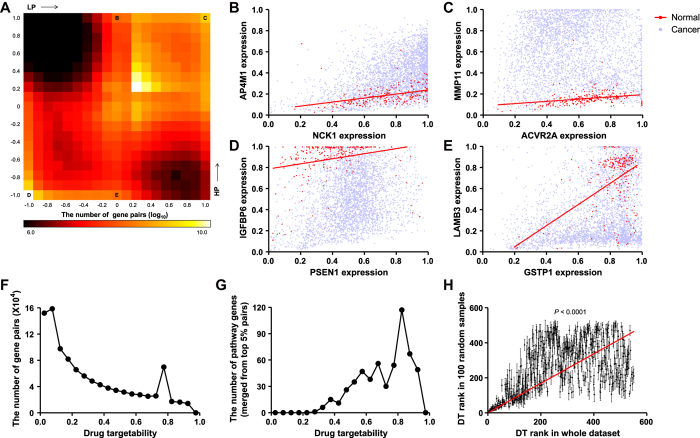
Evaluation of drug targetability in breast cancer. (**A**) Two-dimensional heat map histogram for the number of pathway gene-target gene pairs (log_10_) in terms of LP and HP. (**B**–**E**) Scatter plots of the expression of pathway genes (NCK1, ACVR2A, PSEN1, and GSTP1, respectively) and their target genes (AP4M1, MMP11, IGFBP6, and LAMB3, respectively). (**B**) represents the case of high HP, (**C**) represents the case of both high LP and HP, (**D**) represents the case of both low LP and HP, and (**E**) represents the case of low HP, as indicated in (**A**). (**F**) Frequency distribution of the value of drug targetability for all pathway gene-target gene pairs. (**G**) Frequency distribution of the merged drug targetability value for each pathway gene. The values were merged from pathway gene-target gene pairs with top 5% of drug targetability. (**H**) X-axis indicates the ranks of drug targetability from the whole dataset and y-axis indicates those from 100 random samples. The slope of linear regression (red line) was significantly non-zero (*t* = 44.78). Pearson correlation of two ranks was statistically significant (*P* < 0.0001, R^2^ = 0.3084).

Among many applicable phenotypes, we first examined drug targetability for breast cancer. For all pathway gene-target gene pairs, we calculated LP and HP, and displayed them using a 2D heat map histogram (Fig. 3A). Even though many pairs existed in the center region with a normal transcriptional response, many other pairs showed high LP or HP, implying an abnormal transcriptional response. Several representative examples are shown in Fig. 3. For the cases of C or D in the heat map, target gene expressions were constitutively high (Fig. 3C) or low (Fig. 3D) for cancer. Even though these pairs showed abnormalities, ACVR2A or PSEN1 could not be good drug targets because their target genes still showed abnormal expression levels when their expression was low. However, for the cases of B or E in the heat map, target gene expression in cancer was abnormal only in the high region (Fig. 3B,E). If we inhibit expression of NCK1 or GSTP1 in cancer, we can expect their target gene expression to be similar to the control.

Figure 3F shows the frequency distribution of drug targetability for all pathway gene-target gene pairs. To utilize high-ranking pathway genes as novel drug targets, we averaged drug targetability of each pair into a single value. Therefore, one pathway gene has one value for drug targetability. To highlight abnormal pathway gene-target gene pairs, we used the top 5% pairs for each pathway gene in the merging process. Frequency distributions of these merged drug targetability (finally used values) showed a pattern similar to those of each pair (Fig. 3G). The entire list of drug targetability values for each gene is provided in Supplementary Data S1. We also overlaid these values in the cancer pathway from UnitPath to obtain a systematic view of drug targetability. In the whole pathway diagram, we can easily select drug targetable genes (Supplementary Fig. S2).

To confirm the robustness of this method, we conducted 100 random samplings, and compared drug targetability ranks from whole datasets and those from random samples. The ranks showed statistically significant Pearson correlation, especially in the high rank, indicating the robustness of drug targetability (Fig. 3H).

### Experimental validation of drug targetability

**Figure 4:**
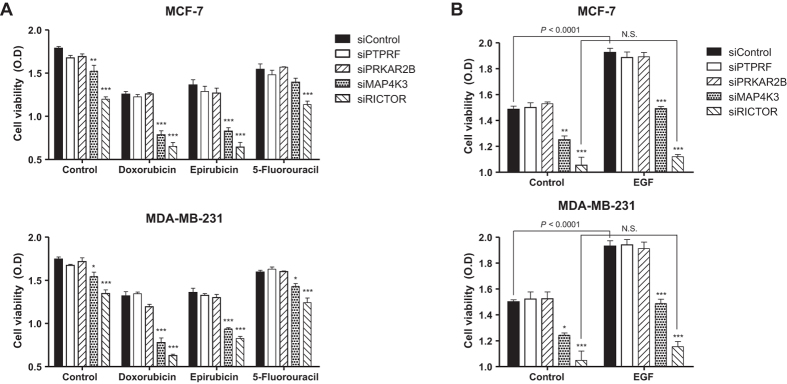
Evaluation of cell viability with knockdown of target genes. Knockdown of PTPRF, PRKAR2B, MAP4K3, and RICTOR was enabled by siRNA transfection as described in the *Materials and Methods*. (**A**) At 48 h post-transfection, cells were treated with doxorubicin (5 mg/L), epirubicin (5 mg/L), and 5-fluorouracil (50 μM) for 24 h, and then cell viability was measured by WST-1 assay. (**B**) At 48 h post-transfection, cells were incubated with EGF or serum-free media for 24 h, and then cell viability was measured by WST-1 assay. Data are presented as means ± SEM, n = 4. Each group (such as Control, Doxorubicin, and EGF) was compared by one-way ANOVA with Tukey’s *post hoc* test for multiple comparisons. For all groups, *P* < 0.0001 in ANOVA, and asterisks indicate significant differences by Tukey’s *post hoc* test (*P < 0.05, **P < 0.01, ***P < 0.001) compared with the siControl. Levels of significance for comparisons between the control and EGF groups in (**B**) were determined using Student’s *t*-test (N.S. indicates not significant).

To verify the accuracy of the computational prediction, we sorted all pathway genes according to drug targetability and selected four top-ranking genes, namely, PTPRF, PRKAR2B, MAP4K3, and RICTOR. After knockdown of these genes, we evaluated cell viability in the presence or absence of several anticancer drugs. In both MCF-7 and MDA-MB-231 cells, knockdown of MAP4K3 or RICTOR reduced cell viability and sensitized anticancer drugs (doxorubicin, epirubicin, and 5-fluorouracil)-induced cell death (Fig. 4A). On the other way, we induced cell proliferation by epidermal growth factor (EGF) treatment in serum-free conditions. As a result, knockdown of MAP4K3 or RICTOR neutralized the proliferative effect of EGF in both cell lines (Fig. 4B). These results suggest that MAP4K3 and RICTOR are responsible for proliferation and growth in breast cancer cells.

**Figure 5:**
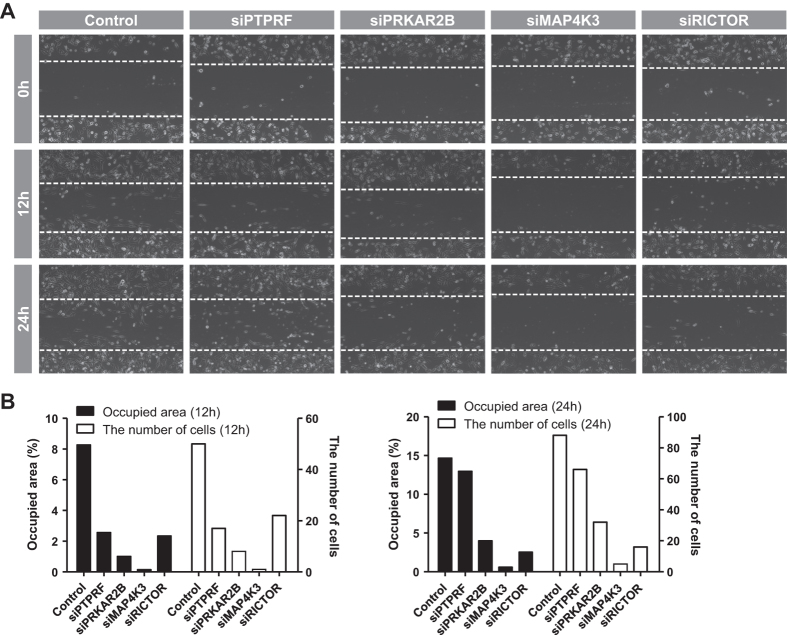
Evaluation of cell migration with knockdown of target genes. Knockdown of PTPRF, PRKAR2B, MAP4K3, and RICTOR was enabled by siRNA transfection, and cell migration was measured by wound-healing assay as described in the *Materials and Methods*. (**A**) At 48 h post-transfection, MDA-MB-231 cells were subjected to a wound-healing assay. Inside the horizontal axis (white dashed line) represents the wounded region. Images were captured at 0, 12, and 24 h using phase contrast microscopy. (**B**) Cell migration was quantified in terms of two features using CellProfiler. Black bar indicates the ratio of the occupied area with migrated cells to the total wounded area. White bar indicates the total number of migrated cells in the wounded region.

We also examined cell migration, which is another hallmark of cancer. At 48 h after transfection of each siRNA, the MDA-MD-231 cells were applied to the wound-healing assay as described in the *Materials and Methods*. Compared to the control, cell migration was remarkably reduced after knockdown of PRKAR2B, MAP4K3, or RICTOR. Especially, MAP4K3 knockdown almost totally blocked cell migration (Fig. 5A). These results were confirmed by quantification, using occupied area and the number of cells in the wounded region (Fig. 5B). To examine transcriptional influence of suggested drug targets, we performed cDNA microarray experiment as direct validation. After knockdown of RICTOR with siRNA, mRNA expression profile was compared to the control. Because our method is aimed to identify pathway genes whose target genes become similar with normal after inhibition, we compared fold changes of normal: cancer (dataset) to those of siRICTOR: control (microarray data). Using 20% as cutoff value in microarray data (fold change, 1.2 < or 0.8>), they have statistically significant Pearson correlation (R = 0.6836, *P*-value = 0.0204). These data suggest that inhibition of RICTOR makes expression levels of its target genes similar with normal samples, validating our method.

**Figure 6:**
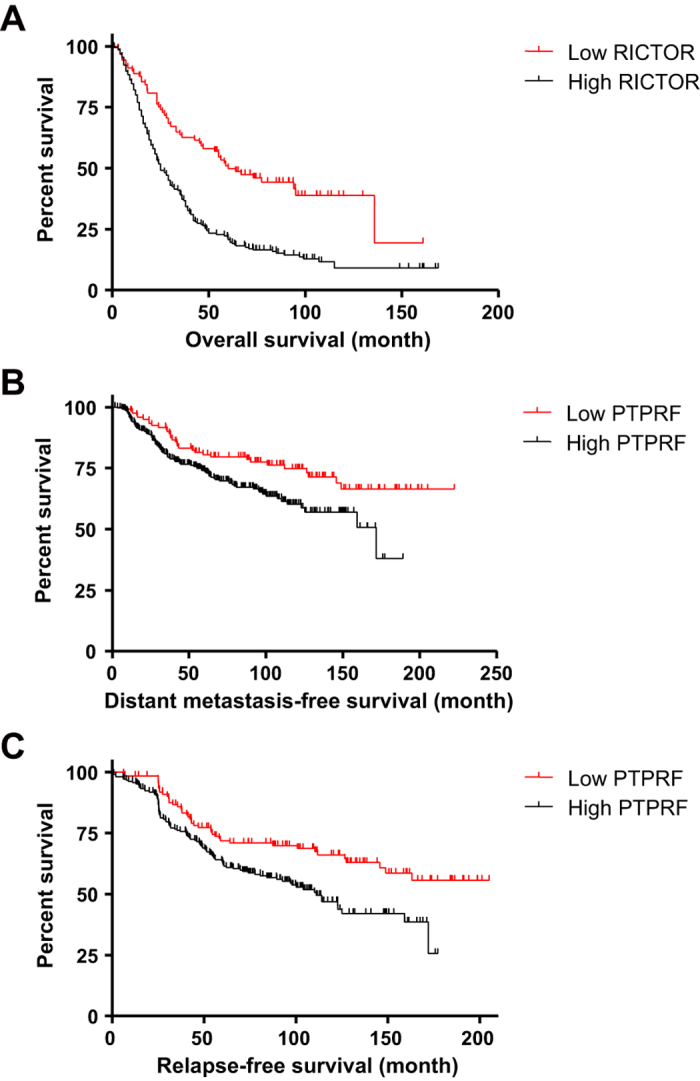
Kaplan-Meier survival analysis for grouped datasets. The Whole breast cancer dataset was divided into two groups according to the expression levels of drug target genes (PTPRF, PRKAR2B, MAP4K3, and RICTOR). Grouped datasets were subjected to Kaplan-Meier survival analysis to compare DMFS, RFS, and OS. Significantly different survival curves according to log-rank test are shown (for (**A**,**B**,**C**), *P* < 0.0001, *P* = 0.0096, and *P* = 0.023, respectively).

With successful experimental validation, we performed Kaplan-Meier survival analysis using patient information annotated in each microarray data. First, we divided whole breast cancer dataset into low or high expression levels of specific drug target genes, and then evaluated distant metastasis-free survival (DMFS), relapse-free survival (RFS), and overall survival (OS) for each grouped dataset. As a result, low RICTOR patients showed significantly longer OS (Fig. 6A), and low PTPRF patients showed significantly longer DMFS (Fig. 6B) and RFS (Fig. 6C), respectively. Even though all other cases were not statistically significant, these data suggest that inhibition of those genes is expected to improve clinical outcomes and prolong lifespan of breast cancer patients.

## Discussion

Many scientists have attempted to develop effective methods for systematic identification of functional driver genes for various diseases. In our previous research, we also successfully showed that the degree of transcriptional response can highlight deterministic genes for different phenotypes^8^. However, reversal of most of these abnormalities with clinical medication is difficult. For a practical application, identification of abnormal genes has proven to be insufficient. In this context, this study is aimed to identify simply restorable genes as useful drug targets.

Extensive selection of effective drug targets is important for targeted therapy in the post-genomic era. To achieve this, we classified and evaluated several different modes of abnormal genes. Practically, fine modulation of gene expression or activity is considerably restricted in an actual clinical setting; most available drugs can only reduce the expression or activity of their target genes^20^,^21^. Among the many genes with an abnormal transcriptional response, therefore, we must select one that becomes normal when its expression is lowered. We defined this feature as drug targetability, and applied our method to breast cancer samples. We computationally predicted PTPRF, PRKAR2B, MAP4K3, and RICTOR to be responsible for the cancer phenotype, and each of them was experimentally validated by means of cell death or migration assay.

Several previous studies investigated RICTOR and MAP4K3, both of which are our proposed drug targets for breast cancer. RICTOR, a well-known component of the mTOR complex 2, was shown to promote migration and prevent apoptosis in osteosarcoma cells and prostate cancer^22^,^23^. Goncharova *et al.* also reported that RICTOR mediates migration in mouse embryonic fibroblasts^24^. MAP4K3 was shown to promote migration and invasion of human non-small cell lung cancer^25^, and to be involved in cell growth through activation of mTOR complex 1 pathway^26^. Despite use of different tissue types or contexts, our experimental results were consistent with these reports in terms of cellular functions.

Owing to high-throughput technology, many researchers have attempted to systematically identify abnormal genes for various diseases. However, only a small number of these genes can be utilized as drug targets. Merely identifying abnormal genes is insufficient. Evaluation of diverse modes of abnormality is required. Our method attempted to predict events after inhibition of each gene by calculating drug targetability. Experimental results and survival analyses confirmed the performance of our method. To verify whether the method we propose here can be widely applied to many other diseases, we examined another case. After comparison between tongue cancer and corresponding normal samples, GNA15, PPP2R3A, LAMB3, HSPA2, and MAP4K3 were identified as five top-ranking genes (Supplementary Data S2). Among them, GNA15^27^, LAMB3^28^, HSPA2^29^, and MAP4K3^25^ were previously reported as prognostic markers in several cancer types, indirectly supporting our method. Proposed method is not limited to cancer, but rather is applicable to any case where two different phenotypes are of interest. We anticipate that our approach will eventually improve treatment efficacy of many diseases.

## Materials and Methods

### Microarray datasets and analysis

We downloaded raw CEL files generated using the Affymetrix Human Genome U133 Plus 2.0 Array (GPL570) platform from the public microarray database Gene Expression Omnibus, and normalized them using the Universal exPression Code method (SCAN.UPC package of Bioconductor)^30^. Samples were grouped by normal and cancer according to the annotations in the original GSM files. For microarray experiment, total RNAs were isolated from MCF-7 cells using an RNeasy Mini Kit (Qiagen, Hilden, Germany), and delivered to commercial microarray service (eBiogen, Seoul, Republic of Korea). The assay was performed using Affymetrix human 2.0 ST array.

### Pre-defined pathways and target gene datasets

We used the UnitPath database (unpublished data, www.unitpath.com) whose pathways are well-organized forms and have interactive modularity for computational analysis. Because UnitPath allows users to select one gene of interest and obtain others that influence the selected genes in each pathway, we used all of these pathway units for analysis. In retrieval of each unit, we demarcated it at the TFs as end points. Information after TFs (target genes of each TF) was obtained from MSigDB c3 TFT v4.0. Finally, we mapped all genes, with connections with at least one TF, to their corresponding target genes (Fig. 1B). In total, we included 1,191 pathway genes and 10,305 target genes of pathway genes in the analysis.

### Evaluation of statistical significance

To evaluate a score for statistical significance, we defined P-score according to the *P*-value. The P-score equation has a form as following:
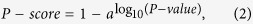
so that the larger *P*-value, the smaller P-score. We excluded multiple test corrections because any cutoff thresholds were not used as significance level in calculation of drug targetability. To project P-score to 0.5 when *P*-value = 0.01, we fixed 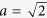. We also added signs to P-score, so that P-score has a minus quantity if the average expression level in cancer is lower than normal; otherwise, P-score is positive. The P-score equation and its relationship with *P*-value are shown in Supplementary Fig. S1.

### Cell cultures and reagents

MCF-7 and MDA-MB-231 cells were maintained at 37 °C in an atmosphere containing 5% CO_2_ in Dulbecco’s modified Eagle’s medium (Welgene, Seoul, Republic of Korea) supplemented with 10% fetal bovine serum (Gibco, Gaithersburg, MD), 100 U/ml penicillin, and 100 μg/ml streptomycin (Invitrogen, Carlsbad, CA). Anticancer drugs (doxorubicin, epirubicin, and 5-fluorouracil) were purchased from Sigma-Aldrich (St.Louis, MO).

### siRNA transfection

A siRNA duplex targeted to PTPRF, PRKAR2B, MAP4K3, RICTOR, and a siRNA with a random sequence (negative control) were synthesized by Bioneer (Daejeon, Republic of Korea). Transient transfection was performed using Lipofectamine 2000 (Invitrogen) according to the manufacturer’s protocol.

### Cell viability assay

WST-1 assays were performed to determine cell viability. MCF-7 and MDA-MB-231 cells were seeded in 24-well plates at a density of 4 × 10^4^ cells/well in quadruplicate, and WST-1 reagent (Nalgene, Rochester, NY) was added to each well up to 5% of the media volume. After incubation for 2 h at 37 °C in a 5% CO_2_ incubator, the absorbance at 450 nm was measured using a microplate reader (Bio-Rad, Richmond, CA). Cell death was confirmed by staining with 100 nM tetramethylrhodamine ethyl ester (TMRE, Sigma-Aldrich). Ten thousand cells were analyzed using a Caliber flow cytometer.

### Wound-healing assay

Adherent MDA-MB-231 cells were scraped from the bottom of the culture surface (six-well plate) using a pipette tip to create a cell-free (wounded) area. Each well was washed with PBS to remove cell debris and then incubated with serum-free media. Several features of cell migration were evaluated using a CellProfiler^31^.

## Additional Information

**How to cite this article**: Park, J. *et al.* Evaluation of drug-targetable genes by defining modes of abnormality in gene expression. *Sci. Rep.* **5**, 13576; doi: 10.1038/srep13576 (2015).

## Supplementary Material

Supplementary Information

Supplementary Information

Supplementary Information
