# Supplementary material for: Evaluation of drug-targetable genes by defining modes of abnormality in gene expression
Source: Sci Rep. 2015 Sep 4;5:13576. doi: 10.1038/srep13576 (PMC4559746; doi:10.1038/srep13576)
Supplement: Supplementary Information [file srep13576-s1.pdf]

Evaluation of drug-targetable genes by defining modes of abnormality in gene expression

Junseong Park*, Jungsul Lee, Chulhee Choi*

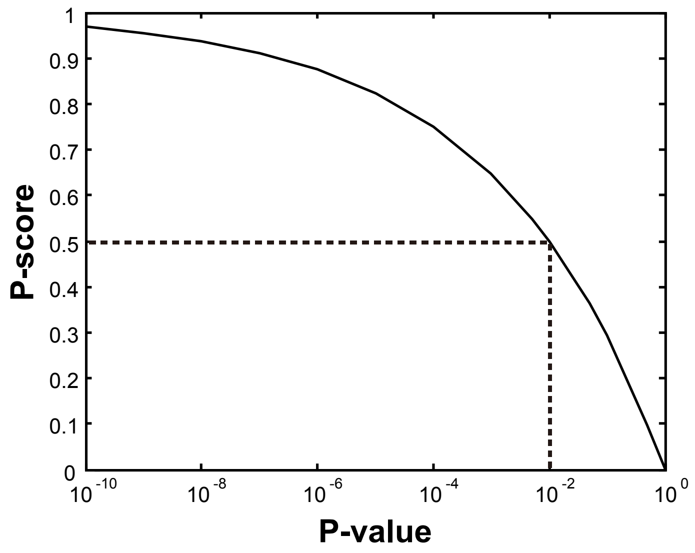

$$P\text{-}score = sign(tg) \times \{1 - \sqrt{2}^{\log_{10}(P\text{-}value)}\}$$

$$sign(tg) = \begin{cases} +1, & \text{if } tg \text{ expression in cancer} \geq \text{in normal} \\ -1, & \text{if } tg \text{ expression in cancer} < \text{in normal} \end{cases}$$

Supplementary Fig. S1. Calculation of P-score according to *P*-value.

P-score is defined by the represented equation. The graph shows relationship between P-score and *P*-value; x-axis indicates *P*-value with log-scale and y-axis indicates P-score. For easy recognition of statistical significance, we projected P-score to 0.5 when *P*-value is 0.01.

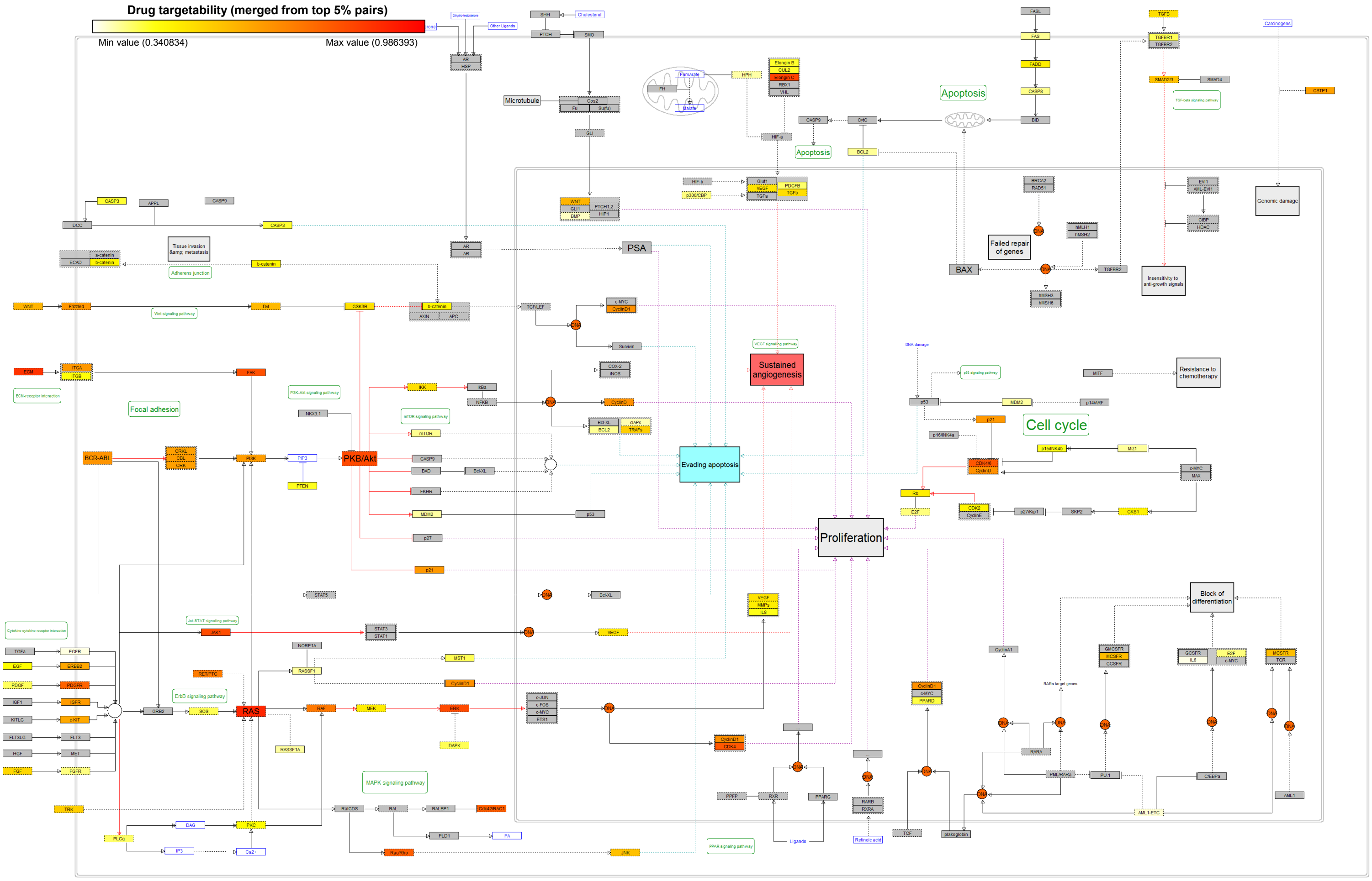

Supplementary Fig. S2. Cancer pathway overlaid with drug targetability values.

In the cancer pathway in the UnitPath database, node colors were overlaid with values of drug targetability. The color map shows the minimum (white) to maximum (red) drug targetability values.
